# Of mice and men: The physiological role of adipose triglyceride lipase (ATGL)^☆^

**DOI:** 10.1016/j.bbalip.2018.10.008

**Published:** 2019-06

**Authors:** Renate Schreiber, Hao Xie, Martina Schweiger

**Affiliations:** Institute of Molecular Biosciences, University of Graz, Austria

**Keywords:** ATGL, PNPLA2, Lipolysis, NLSDM

## Abstract

Adipose triglyceride lipase (ATGL) has been discovered 14 years ago and revised our view on intracellular triglyceride (TG) mobilization – a process termed lipolysis. ATGL initiates the hydrolysis of TGs to release fatty acids (FAs) that are crucial energy substrates, precursors for the synthesis of membrane lipids, and ligands of nuclear receptors. Thus, ATGL is a key enzyme in whole-body energy homeostasis. In this review, we give an update on how ATGL is regulated on the transcriptional and post-transcriptional level and how this affects the enzymes' activity in the context of neutral lipid catabolism. In depth, we highlight and discuss the numerous physiological functions of ATGL in lipid and energy metabolism. Over more than a decade, different genetic mouse models lacking or overexpressing ATGL in a cell- or tissue-specific manner have been generated and characterized. Moreover, pharmacological studies became available due to the development of a specific murine ATGL inhibitor (Atglistatin®). The identification of patients with mutations in the human gene encoding ATGL and their disease spectrum has underpinned the importance of ATGL in humans. Together, mouse models and human data have advanced our understanding of the physiological role of ATGL in lipid and energy metabolism in adipose and non-adipose tissues, and of the pathophysiological consequences of ATGL dysfunction in mice and men.

## Highlights

• Summary of mouse models with genetic or pharmacological manipulation of ATGL.
• Summary of patients with mutations in the human gene encoding ATGL.
• In depth discussion of the role of ATGL in numerous physiological processes in mice and men.

## 1 Introduction

The integrity of an organism depends on the balance between energy uptake and expenditure. If caloric intake exceeds expenditure, the energy surplus is converted into fatty acids (FAs) which are then esterified to glycerol and stored as triglycerides (TGs). When energy expenditure (EE) surpasses caloric intake, stored TGs are hydrolyzed to release FAs in a process called lipolysis. Virtually all mammalian cell types are able to store and mobilize TGs, although in varying quantities and for different purposes. The lipolytic pathway has been revised 14 years ago, when adipose triglyceride lipase (ATGL) was discovered to be the main enzyme responsible for the initial step of TG degradation. Since then, a multitude of different functions and metabolic implications have been ascribed to the enzyme. As a key player in lipid catabolism, ATGL affects whole-body energy homeostasis and thus, it is not surprising that impaired ATGL activity is associated with disease in mice and men. In this review, we discuss the diverse physiological functions of ATGL in different cell types and tissues based on studies (i) in the mouse upon genetic deletion or pharmacological inhibition and (ii) in humans – in particular by the characterization of patients with mutations in the human ATGL gene.

## 2 Update on ATGL biochemistry

In 2004, patatin-like phospholipase domain containing 2 (PNPLA2) was discovered by three independent laboratories and designated ATGL [1], desnutrin [2], and iPLA2ζ [3], respectively. For simplification, we will refer to PNPLA2 as ATGL. ATGL hydrolyzes TG species containing long-chain FAs at the *sn*-2 and *sn*-1, but not at the *sn*-3 position [4]. Additionally, it has been shown that ATGL contributes to retinyl ester (RE) mobilization in hepatic stellate cells [5], and possesses phospholipase A_2_ activity towards [1,2‑dilinoleoyl]‑phosphatidylcholine [6]. Interestingly, Jenkins et al. [3] also detected CoA independent acylglycerol transacylase activity for ATGL generating diglycerides (DG) from two monoglycerides (MG) and TG from MG as acyl-donor and DG as acyl-acceptor. While the TG hydrolase activity is well established, the other enzymatic activities of ATGL and their physiological impact are less characterized. Therefore, we refer to TG hydrolase activity as ATGL activity unless otherwise stated.

**Fig. 1:**
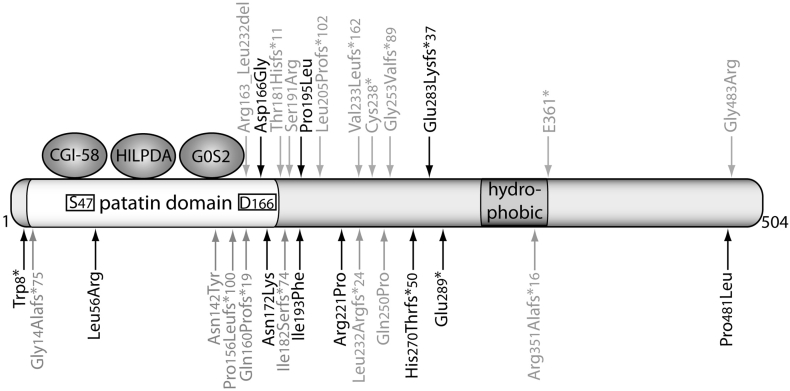
Functional domains of human ATGL. Human ATGL comprises 504 amino acids. The patatin domain contains a catalytic dyad consisting of the active site serine (S47) and the aspartatic acid (D166). A hydrophobic stretch at the C-terminal domain contains the putative LD binding region. CGI-58, HILPDA, and G0S2 directly interact with the patatin domain of the protein. Mutations in the human gene encoding ATGL from diagnosed NLSDM patients are highlighted (i) in black that have been shown to impair LD localization and/or enzymatic activity, or (ii) in grey that have not been characterized for their impact on ATGL function. *, a premature stop occurs immediately or after a frameshift (fs) of several amino acids.

The catalytic site of murine and human ATGL consists of an unusual dyad comprising serine 47 and aspartate 166 located within the patatin domain at the N-terminus of the protein (Fig. 1) [7]. The C-terminal part contains a hydrophobic lipid droplet (LD) binding region [8]. ATGL mainly localizes to TG-rich intracellular LDs [1] and several factors affect its localization at the site of enzymatic action. (i) Most importantly, mutations within the C-terminus of ATGL critically interfere with LD binding [8]. (ii) ATGL binds to LC3, an autophagosomal marker, which increases the recruitment of ATGL to the LD highlighting a crosstalk between neutral lipolysis and lipophagy [9]. (iii) Targeting of ATGL from the endoplasmic reticulum to the LD depends on the vesicular transport machinery and on the direct interaction with Golgi-Brefeldin A resistance factor and coat complex I/II [[10], [11], [12]]. And (iv), more recently, phosphorylation of Thr372 has been shown to prevent LD localization of murine ATGL [13]. While the latter have not been studied in detail concerning physiological settings, mutations leading to a truncated ATGL protein lacking the C-terminus have detrimental physiological consequences in humans (discussed below). Functional domains and regulatory sites of human ATGL are illustrated in Fig. 1.

ATGL protein is expressed at low levels in several non-adipose tissues like skeletal muscle, liver, heart, testes [1], lung [14], retina [6], immune cells [[15], [16], [17]], pancreas [18], and small intestine [19], but is highly expressed in white and brown adipose tissue (WAT and BAT, respectively). ATGL expression/activity is regulated on transcriptional and post-transcriptional levels. Transcriptionally, during adipocyte differentiation, peroxisome proliferator-activated receptor gamma (PPAR-γ) interacts with specificity protein 1 at the ATGL promoter and induces ATGL mRNA expression [20,21]. Fat specific protein 27 (Fsp27) and insulin modulate the affinity of early growth response protein 1 to the ATGL promoter thereby reducing ATGL transcript levels [22,23]. Binding of the transcriptional activators interferon regulatory factor 4 [24] and forkhead box protein O1 (FoxO1) to the ATGL promoter is increased upon fasting [25] and reduced by insulin [24,26,27]. Recently, it has been shown that a long non-coding RNA, steroid receptor RNA activator, represses FoxO1 as well as PPAR-γ mediated ATGL transcription, although by a yet unknown mechanism [28]. ATGL mRNA expression is positively regulated by STAT5 linking the response of adipocytes to growth hormones to an increase in ATGL-mediated lipolysis [29]. In accordance with induced mRNA expression, ATGL protein levels increase in adipose tissues upon fasting [30]. Interestingly, β-adrenergic stimulation reduces transcript levels of ATGL while its activity is tremendously increased highlighting the importance of post-transcriptional regulation of lipolysis [1,31]. Post-transcriptionally, ATGL is modified by phosphorylation. Murine ATGL consists of putative phosphorylation sites at Ser87, Thr101, Thr210, Thr372, Tyr378, Ser393, Ser406, and Ser430 [13,32]. Phosphorylation at Ser406 either by protein kinase A (PKA) [33] or by AMPK [34,35] increases murine ATGL activity. As a feedback regulatory mechanism, long chain acyl-CoAs bind to the N-terminal part of ATGL and inhibit its activity in vitro indicating a product inhibition of ATGL [36]. Moreover, in adipocytes and hepatocytes, ATGL is ubiquitinated and targeted for proteasomal degradation [37,38] suggesting that ATGL activity is also regulated by modulating protein stability.

Similar to other lipases, ATGL activity is highly regulated by several cofactors. Best studied is comparative gene identification-58 (CGI-58; also known as α/β hydrolase domain containing 5, ABHD5) that directly binds to and activates ATGL activity [39] and broadens its regio-specificity from *sn*-2 only to *sn*-1 and *sn*-2 [4]. Interestingly, binding of adipocyte-fatty acid binding protein to CGI-58 further induces ATGL activity, presumably by preventing product inhibition [40]. In non-adipocytes, UBXD8 interacts with ATGL and recruits a segregase which forces dissociation of CGI-58 to reduce ATGL activity [41]. LD associated proteins of the perilipin family were shown to regulate lipolysis by withholding CGI-58 from ATGL under basal, and releasing CGI-58 to facilitate ATGL activation under lipolytic stimulated conditions [[42], [43], [44], [45]]. As an antipode for CGI-58, G0/G1 switch gene 2 (G0S2) potently inhibits ATGL activity by direct protein-protein interaction and through impeding substrate accessibility [46]. Recently, it has been shown that ATGL is profoundly inhibited by a small protein called hypoxia-inducible LD-associated protein (HILPDA, also known as hypoxia inducible gene 2, HIG2) which directly binds to the patatin domain of ATGL [47,48]. Another player in the team of ATGL regulators is pigment epithelium-derived factor (PEDF). By binding to ATGL, PEDF increases phospholipase activity of ATGL in a dose dependent manner in vitro [6] and mediates an anti-apoptotic effect of ATGL in retina cells [49]. Although the mechanism is yet not clear, PEDF has been shown to stimulate TG hydrolase activity in liver and muscle lysates in vitro and to increase the release of FAs from murine adipocytes and adipose tissues in an ATGL dependent manner [50].

In humans, ATGL is also activated by CGI-58 [39,51] and inhibited by G0S2 [52] and HILPDA [48] (Fig. 1). Yet, species-specific differences exist: (i) Activation of ATGL activity by CGI-58 is less pronounced in humans than in mice [39], and (ii) the C-terminus of human but not murine ATGL impairs its activity [8]. In human adipocytes, Fsp27 reduces ATGL-mediated lipolysis in vivo, however no direct effect was observed in vitro [53].

## 3 What we have learnt from mouse models and patients

Since the discovery of ATGL in 2004, different genetic knockout and transgenic mouse models have provided important insights into the (patho)-physiological function of ATGL. The recent development of an ATGL specific inhibitor (Atglistatin® [54]) allowed acute and chronic pharmacological inhibition of ATGL and enabled comparisons to genetic models. A comprehensive list of published ATGL mouse models and associated phenotypes is given in Table 1. In humans, >40 patients with mutations in the human gene encoding ATGL are identified to date [[55], [56], [57], [58]]. Progressive (cardio)-myopathy and TG accumulation in leukocytes and multiple tissues characterizes these patients. Consequently, this rare autosomal recessive disease was designated as neutral lipid storage disease (NLSD) with myopathy (NLSDM) [59]. The severity of the disease depends on the number of affected alleles (homozygous vs. heterozygous), the affected sites within the protein, and whether the mutation impairs enzyme activity and/or LD localization. To date, few functional analyses were performed in humans due to the limited number of affected individuals and thus, the accessibility of patients. A list of published NLSDM patients and mutant carriers including genetics, biochemistry, and pathophysiology is provided in Table 2. Moreover, Fig. 1 depicts the amino acid positions of the respective mutations associated with NLSDM within the human ATGL protein. In the following sections, we will discuss what we have learnt about ATGL function over more than a decade in mice and men.

**Table 1:** Summary of mouse models with genetic ATGL modifications.

Genotype	Synonyms	Knockout/transgene	Promotor	Phenotype	References
Systemic ATGL knockout	AKO	Knockout	–	Mild obesity, cardiomyopathy, premature lethality, age-dependent cold sensitivity, impaired VLDL secretion	[62,65]
Systemic ATGL knockout/heart-rescued	AKO/cTg, ATGL-ko/CM, *Atgl*KO-cm*Atgl*TG, Atgl-/-ctg	Knockout/transgene	-/α-MHC	Mild obesity (chow), obesity resistance (HFD), improved glucose homeostasis (GTT, ITT, clamp)	[61,65,71,77]
Adipocyte-specific ATGL knockout	AAKO	Knockout	AdiQ-Cre	Mild obesity (chow), ~obesity (HFD), improved GTT and ITT (chow + HFD), AT inflammation, impaired exercise, impaired insulin and VLDL secretion, cold tolerant in ad lib fed state and intolerant upon fasting	[64,65,113,165,179]
Adipocyte-specific ATGL knockout	ASKO, ATGLAKO	Knockout	aP2-Cre	Mild obesity (chow), aggravated obesity (HFD), fasting/cold-induced hypothermia, improved glucose homeostasis (GTT, ITT, clamp); cold intolerant upon fasting	[35,63]
Adipocyte-specific ATGL overexpression	aP2-desnutrin	Transgene	aP2	Obesity resistant (HFD), increased EE, improved glucose homeostasis (clamp)	[81]
BAT-specific ATGL knockout	iBAKO	Knockout	UCP-1-CreER	BAT hypertrophy, cold tolerant	[65]
Adipocyte-specific ATGL and HSL knockout	DAKO	Knockout	aP2	Cold intolerant upon fasting; liposarcoma	[183]
WT overexpressing ATGL	MHC-ATGL	Transgene	α-MHC	Prevents cardiac dysfunction upon metabolic disorders	[157]
Cardiomyocyte-specific ATGL knockout	iHAKO	Knockout	α-MHC-CreER	Cardiomyopathy, fibrosis, inflammation, age-dependent cold sensitivity	[65,149]
Skeletal muscle-specific ATGL knockout	SMAKO	Knockout	Myo	Increased IMTG, normal exercise performance, no systemic effect	[118,165]
Skeletal muscle-specific ATGL transgene	Tg	Transgene	Muscle creatine kinase	Decreased IMTG, no systemic effect	[118]
Hepatocyte-specific ATGL knockout	ATGLLKO	Knockout	Alb-Cre	Hepatic TG accumulation, normal VLDL secretion, GTT, ITT, and PTT	[117]
β-cell-specific ATGL knockout	βKO	Knockout	RIP-Cre/RIP-CreER	Impaired GSIS, improved GTT, normal ITT	[76]
β-cell-specific ATGL knockout	B-*Atgl*-KO	Knockout	Mip-CreERT	Impaired GSIS, normal GTT	[114]

**Table 2:** Summary of diagnosed NLSDM patients and affected mutant carriers. Mutated proteins which have been confirmed to harbor biochemical defects in regard to ATGL activity and/or ATGL localization at the LD are highlighted in bold.

Recessive alleles	Nucleotide change	Amino acid change	Affected site of protein	ATGL activity	ATGL localization	Cardio-myopathy	Myopathy	CK levels	Liver dysfunction	Obesity	Glucose metabolism	Others	TG accumulation	References
Homozygous	c.497A > G	**p**.**Asp166Gly**	Catalytic dyad	No	Yes	^+++^ CTx	n.i.	n.i.	n.i.	n.i.	n.i.	n.i.	n.i.	[67]
Compound heterozygous	c.497A > G c.1442C > T	**p**.**Asp166Gly** **p**.**Pro481Leu**	Catalytic dyad C-term.	No yes	Yes yes	^−^	^+^	^+^	^−^	n.i.	n.i.	n.i.	L	[150]
Homozygous (both)	c.584C > T c.1447G > C	**p**.**Pro195Leu** p.Gly483Arg	N-term. C-term.	No n.d.	Yes n.d	^+++^	^++^	^+++^	^−^	^−^	^+++^	^−^	L, M	[128]
Compound heterozygous	het c.584C > T c.808_808delC	**p**.**Pro195Leu** **p**.**His270Thrfs** ∗ **50**	N-term. C-term.	No yes	Yes no	^+^	^++^	^+^	^++^	^−^	^−^	Short stature, recurrent infections, hearing loss, psoriasis	L, M	[8,59,120,126]
Heterozygous	c.584C > T	**p**.**Pro195Leu**	N-term.	No	Yes	n.i.	^+^	^−^	n.i.	n.i.	n.i.	Recurrent infections	L	[126]
Heterozygous	c.584C > T	**p**.**Pro195Leu**	N-term.	No	Yes	^−^	^+^	n.i.	^−^	n.i.	^−^	^−^	L	[126]
Heterozygous	c.808_808delC	**p**.**His270Thrfs** ∗ **50**	C-term.	Yes	No		^+^	^−^	^−^	^+^	n.i.	Exercise intolerance	L, M	[120]
Heterozygous	c.808_808delC	**p**.**His270Thrfs** ∗ **50**	C-term.	Yes	No	^−^	^−^	n.i.	^−^	n.i.	n.i.	Hearing loss, psoriasis	L	[126]
Heterozygous	c.808_808delC	**p**.**His270Thrfs** ∗ **50**	C-term.	Yes	No	^−^	^+^	^−^	^−^	n.i.	^−^	Recurrent infections	L, M	[126]
Heterozygous	c.808_808delC	**p**.**His270Thrfs** ∗ **50**	C-term.	Yes	No	^−^	^−^	^−^	^−^	n.i.	^−^	Recurrent infections	L	[126]
Heterozygous	c.808_808delC	**p**.**His270Thrfs** ∗ **50**	C-term.	Yes	No	^−^	^−^	^−^	^−^	n.i.	^−^	Recurrent infections	L	[126]
Homozygous	c.847_847delC	**p**.**Glu283Lysfs** ∗ **37**	C-term.	Yes	No	^+++^	^+^		^++^	^−^	^+++^	Mental retardation, short stature, intestinal problems, pancreatitis	L, M	[59]
Homozygous	c.865C > T	**p**.**Glu289**^⁎^	C-term.	Yes	No	^+^	^++^	^+++^	^+^	^−^	^−^	^−^	L, M	[8,59,220]
Homozygous	hom. c.865C > T	**p**.**Glu289**^⁎^	C-term.	Yes	No	^+++^ CTx	^+^	n.i.	n.i.	n.i.	n.i.	^−^	L, M, H	[79,221]
Compound heterozygous	c.865C > T c.424A > T	**p**.**Glu289**^⁎^ p.Asn142Tyr	C-term. N-term.	Yes n.d.	No n.d.	^−^	^−^	^+++^	^−^	^−^	^++^	^−^	L, M	[127]
Compound heterozygous	c.865C > T c.424A > T	**p**.**Glu289**^⁎^ p.Asn142Tyr	C-term. N-term.	Yes n.d.	No n.d.	^+++^	n.d.	n.d.	n.d.	n.d.	n.d.	^−^	n.d.	[119]
Compound heterozygous	c.24G > A c.516C > A	**p**.**Trp8**^⁎^ **p**.**Asn172Lys**	Potential null allele patatin dom.	n.d. low	n.d. yes	^+^	^+++^	^++^	n.i.	n.i.	n.i.	^−^	L, M	[222]
Compound heterozygous	c.24G > A c.516C > A	**p**.**Trp8**^⁎^ **p**.**Asn172Lys**	Potential null allele patatin dom.	n.d. low	n.d. yes	^−^	^+++^	^++^	n.i.	n.i.	n.i.	^−^	L	[222]
Homozygous	c.662G > C	**p**.**Arg221Pro**	N-term.	Low	Yes	^−^	^+^	^+++^	n.i.	n.i.	^−^	Pancreatitis	L	[222]
Homozygous	c.662G > C	**p**.**Arg221Pro**	N-term.	Low	Yes	^−^	^+++^	^++^	n.i.	n.i.	^−^	Chronic diarrhea	L	[22]
Compound heterozygous	c.177 T > G c.577A > T	**p**.**Leu56Arg** **p**.**Ile193Phe**	Close to catalytic dyad N-term.	Low low	Yes yes	^−^	^++^	^++^	^++^	n.i.	n.i.	^−^	L, M	[56]
Compound heterozygous	c.177 T > G c.577A > T	**p**.**Leu56Arg** **p**.**Ile193Phe**	Close to catalytic dyad N-term.	Low low	Yes yes	^−^	^+^	^++^	^+^	n.i.	n.i.	^−^	L, M	[56]
Compound heterozygous	c.177 T > G c.577A > T	**p**.**Leu56Arg** **p**.**Ile193Phe**	Close to catalytic dyad N-term.	Low low	Yes yes	−	^+^	^+^	^+^	n.i.	^+++^	Insulin therapy, hyperTG	L, M	[56]
Homozygous	c.695_695delT	p.Leu232Argfs ∗ 24	C-term.	n.d.	n.d.	^−^	^+++^	^++^	^+^	^−^	^+++^	Hyperlipidemia	L, M, VAT	[68,167]
Homozygous	c.695_695delT	p.Leu232Argfs ∗ 24	C-term.	n.d.	n.d.	^−^	^+^	^++^	^−^	^−^	^−^	^−^	L, M	[128]
Homozygous	c.541_542delAC	p.Thr181Hisfs ∗ 11	N-term.	n.d.	n.d.	^+++^	^++^	^++^	^+^	^−^	^−^	^−^	L, M, P, VAT	[68,167]
Homozygous	c.541_542delAC	p.Thr181Hisfs ∗ 11	N-term.	n.d.	n.d.	^+^	^+^	^++^	^+^	n.i.	^−^	Hyperlipidemia	L, M, P, H, VAT	[68,167]
Homozygous	c.477_478insCCTC	p.Gln160Profs ∗ 19	Patatin dom.	n.d.	n.d.	^−^	^+++^	^+^	^−^	n.i.	^−^	^−^	L, M	[223]
Homozygous	c.477_478insCCTC	p.Gln160Profs ∗ 19	Patatin dom.	n.d.	n.d.	^+^	^+^	^+^	^−^	n.i.	n.i.	^−^	L, M	[166]
Homozygous?	c.477_478insCCTC	p.Gln160Profs ∗ 19	Patatin domain	n.d.	n.d.	^+^	^+^	^++^	^−^	n.i.	n.i.	^−^	L, M	[168]
Homozygous?	c.477_478insCCTC	p.Gln160Profs ∗ 19	Patatin domain	n.d.	n.d.	^+^	^+^	^+^	^−^	n.i.	n.i.	^−^	L, M	[168]
Homozygous	c.187 + 1G > A	n.i.	Splice donor site	n.d.	n.d.	^+^	^+^	n.i.	n.i.	n.i.	n.i.	Hearing loss, short stature	M	[224]
Homozygous	c.187 + 1G > A	n.i.	Splice donor site	n.d.	n.d.	^−^	^+^	n.i.	n.i.	n.i.	n.i.	^−^	M	[224]
Homozygous	c.612_613insC	p.Leu205Profs ∗ 102	N-term.	n.d.	n.d.	^−^	^+^	^+++^	^+^	^++^	n.i.	HyperTG	L, M	[128]
Homozygous	c.612_613insC	p.Leu205Profs ∗ 102	N-term.	n.d.	n.d.	^−^	^+^	^+++^	^+^	^+^	n.i.	Mild hyperTG	L, M	[128]
Homozygous	c.1051_1051delC	p.Arg351Alafs ∗ 16	C-term. (hydrophobic stretch)	n.d.	n.d.	^−^	^+^	^+++^	^−^	^+++^	n.i.	HyperTG	L, M	[128]
Homozygous	c.543_543delC	p.Ile182Serfs ∗ 74	N-term.	n.d.	n.d.	^+^	^+^	^++^	^−^	^−^	n.i.	Hearing loss	L, M	[128]
Homozygous	c.1081G > T	p.E361^⁎^	C-term.	n.d.	n.d.	n.i.	^+^	^−^	n.i.	n.i.	n.i.	^−^	L	[169]
n.i.	Retrotransposon	n.i.	N-term.			^−^	^−^	^+++^	^+^			Malar flush	L, M, H	[225]
Homozygous	c.696 + 1G > C	p.Val233Leufs ∗ 162 and p.Arg163_Leu232del	C-term. and catalytic dyad	n.d.	n.d.	^+++^ Ctx	n.i.	n.i.	n.i.	n.i.	n.i.	^−^	L, H, M	[79]
Compound heterozygous	c.757 + 2 T > C c.749A > C	n.i. p.Gln250Pro	Splice donor site N-term.	n.d.	n.d.	^−^	^++^	^+^	^−^	n.i.	^−^	^−^	L, M	[226]
Homozygous	c.467_467delC	p.Pro156Leufs ∗ 100	Patatin dom.	n.d.	n.d.	^−^	^+++^	^+++^	^−^	n.i.	^−^	^−^	L, M	[226]
Homozygous	c.757 + 1G > T	p.Gly253Valfs ∗ 89	Splice donor site	n.d.	n.d.	^−^	^+++^	^+++^	^−^	n.i.	^−^	^−^	L, M	[226]
Homozygous	c.757 + 1G > T	p.Gly253Valfs ∗ 89	Splice donor site	n.d.	n.d.	^−^	^+++^	^++^	^−^	n.i.	^−^		L, M	[58]
Homozygous	c.757 + 1G > T	p.Gly253Valfs ∗ 89	Splice donor site	n.d.	n.d.	^−^	^++^	^+^	^−^	n.i.	^−^	Hearing loss	L, M	[58]
Homozygous	c.757 + 1G > T	p.Gly253Valfs ∗ 89	Splice donor site	n.d.	n.d.	^−^	^+^	^+^	^−^	n.i.	^−^		L, M	[58]
Homozygous	c.757 + 1G > T	p.Gly253Valfs ∗ 89	Splice donor site	n.d.	n.d.	^−^	^+^	^+++^	^+^	n.i.	^−^	Short stature, hyperTG	L, M	[58]
Homozygous	c.757 + 1G > T	p.Gly253Valfs ∗ 89	Splice donor site	n.d.	n.d.	n.i.	^+^	^+++^	n.i.	^−^	n.i.	^−^	M	[227]
Homozygous	c.757 + 1G > T	p.Gly253Valfs ∗ 89	Splice donor site	n.d.	n.d.	^+^	^+^	^+++^	^+^	^+^	^−^	^−^	L, M	[220]
Homozygous	c.757 + 1G > T	p.Gly253Valfs ∗ 89	Splice donor site	n.d.	n.d.	^−^	^+^	^+++^	^−^	^−^	^−^	^−^	L, M	[220]
Homozygous	c.571A > C	p.Ser191Arg	N-term.	n.d.	n.d.	^+^	^++^	^+^	n.i.	n.i.	^+++^	Hearing loss	L, M	[228]
Homozygous	c.714C > A	p.Cys238^⁎^	N-term	n.d.	n.d.	^+^	^++^	^+++^	^+^	n.i.	^+^	Cognitive impairment, hearing loss, mild hyperTG, intestinal symptoms	L, M	[229]
Homozygous	c.41_47delGCTGCGG	p.Gly14Alafs75^⁎^	N-term	n.d.	n.d.	^+++^	^++^	^+++^	n.i.	n.i.	n.i.	Exercise intolerance	L,M	[170]

Mutations are indicated according to the recommendations from the Human Genome Variation Society. Definition of diseases: Obesity including overweight; Impaired glucose metabolism as assessed by hyperinsulinemic/euglycemic clamp, oral glucose tolerance, and/or insulin secretion; Liver dysfunction includes hepatomegaly, hepatosteatosis, increased plasma alanine aminotransferase, and aspartate aminotransferase levels; Severity grading of disease: −, no disease observed; +, mild; ++, moderate; +++, severe; Abbreviations/indices: n.i. = not identified; n.d. = not determined; L, leucocytes; M, muscle; P, pancreas, H, heart; hyperTG, hypertriglyceridemia; VAT, visceral adipose tissue; CTx, cardiac transplant (obtained/awaiting).

### 3.1 ATGL and energy homeostasis

i) Energy supply via ATGL

To meet the energy requirements of the body, FA release from adipose tissues is low after a meal and high upon fasting. Accordingly, ATGL-mediated lipolysis in adipocytes correlates with the body's energy status and is regulated by the aforementioned transcriptional and post-transcriptional mechanisms. In mice, ATGL is highly expressed in adipose tissues [1] and together with hormone-sensitive lipase (HSL) represents one of two major TG lipases [60]. Thus, adipocyte ATGL controls whole-body FA and energy supply dependent on the nutritional status. In the ad libitum fed state, the systemic loss of ATGL did not impair EE [61,62] but the loss of ATGL specifically in adipocytes moderately increased the respiratory exchange ratio (RER) and food intake [63,64]. These observations indicate that ATGL deficient mice preferentially utilize energy substrates provided by the food. Upon high energy requirements like fasting, cold exposure, or exercise, ATGL-mediated lipolysis in WAT becomes limiting and the loss of ATGL in WAT causes a depletion of endogenous and circulating energy substrates provoking a hypo-metabolic state and hypothermia [35,[61], [62], [63], [64], [65]]. Loss of ATGL in adipocytes causes a faster sympathetic activation of WAT [63], presumably to allow FA release via HSL-mediated TG mobilization from WAT.

In humans, the contribution of ATGL to adipocyte lipolysis and whole-body energy metabolism is less clear. Similar as in mice, ATGL and HSL are the major lipases in adipocytes [52]. However, whether or not ATGL-mediated lipolysis contributes to basal and catecholamine-stimulated lipolysis is still under debate [51,66]. A population-wide association study showed non-significant effects of rare ATGL variants on plasma FA levels [67]. Furthermore, while norepinephrine infusion studies confirmed the lipolytic defect in NLSDM patients, fasting plasma FA levels, glycerol turnover rates, and RER values were similar in patients and healthy controls [68].

ii) ATGL affects mitochondrial FA oxidation

FA oxidation is transcriptionally controlled by PPARs that are members of the superfamily of nuclear hormone receptors [69,70]. Three distinct isoforms exist that show a characteristic tissue expression pattern: PPAR-α is highly expressed in oxidative tissues like heart, skeletal muscle, liver, and BAT. PPAR-δ is expressed in many cells, whereas PPAR-γ is most abundantly expressed in adipocytes. PPARs are ligand-activated transcription factors and FAs are their bona fide ligands [69]. In line with the key role of ATGL in FA release, any modulation of ATGL activity directly and/or indirectly affects PPAR activation: PPAR-α in the heart [71], BAT [35,72], liver [63,64,73], and macrophages [74,75], most likely PPAR-δ in the pancreatic β-cell [76], and PPAR-γ in WAT [77,78]. Pathophysiological consequences of ATGL loss are most severe in the heart leading to premature lethality in mice [62,71] and humans [67,79] (Table 2). A detailed discussion of the role of PPARs is included in the respective sections.

### 3.2 Impact of ATGL on obesity and adipose tissue inflammation

The world health organization defines obesity as abnormal or excessive fat accumulation that may impair health. Obesity develops upon a dysregulated energy balance when more calories are consumed than expended. As Hippocrates claimed: “Obesity is not only a disease by itself, but a harbinger of others”, increased fat mass is associated with several metabolic complications like impaired insulin action, hyperlipidemia, fatty liver, and cardiovascular disease that are summarized in the term “metabolic syndrome” [80]. One of the mechanisms that link hypertrophic adipocytes to metabolic derangements in obesity is adipose tissue inflammation. How obesity development and adipose tissue inflammation are altered upon loss or overexpression of ATGL is discussed below.

i) Feedback regulation of lipolysis and lipid synthesis/storage

Impaired TG mobilization from adipocytes is expected to cause obesity and to aggravate upon nutritional stress. Accordingly, upon chow diet, systemic and adipocyte-specific ATGL knockout mice exhibited moderate obesity with a ~2-fold increase in WAT depots and 5- to 7-fold increase in BAT [[62], [63], [64],^77^,^81^]. In contrast, the obesity grade is somewhat inconsistent upon high-fat diet (HFD) with increased [35] or normal adiposity [64] in adipocyte-specific ATGL knockout mice, or decreased adiposity in systemic ATGL knockout mice with normal cardiac function [77]. However, considering that epididymal WAT mass is hardly different in adipocyte-specific ATGL knockout mice compared to controls [35,64], the phenotype of these mice also point towards a resistance to diet-induced obesity (DIO). Moreover, pharmacological inhibition of ATGL reversed DIO [78]. Remarkably, the obesity-resistant phenotype did not cause ectopic lipid accumulation neither of “inert” TG species nor of so-called bioactive lipids like DGs or ceramides [35,77,78]. Thus, as discussed in the section “Effect of ATGL on glucose homeostasis”, obesity-resistant ATGL knockout mice maintain glucose tolerant and insulin sensitive arguing against a metabolically “lipodystrophic” phenotype.

The obesity resistant phenotype is not restricted to ATGL. Systemic HSL knockout mice are also resistant to DIO [[82], [83], [84]]. Additionally, neither ATGL nor HSL deficient humans are commonly obese [85] (Table 2). Together, these phenotypes indicate a feedback regulatory mechanism of lipolysis and lipogenesis [77,78,86]. Accumulating evidence support the concept that ATGL-/HSL-mediated lipolysis directly or indirectly affect signaling via the key adipogenic/lipogenic transcriptional regulator PPAR-γ leading to attenuated lipid synthesis and storage [77,84,86]. Accordingly, a reconstitution of PPAR-γ signaling via the synthetic agonist rosiglitazone partially reversed the obesity-resistant phenotype in ATGL [77] and HSL knockout mice [87]. Yet, a cell-autonomous defect in adipogenesis was not observed and PPAR-γ transcript and protein levels were only decreased in vivo [77]. Another interesting aspect in mice with genetic and pharmacological inhibition of ATGL is the key anabolic hormone insulin. As discussed below, genetic and pharmacological inhibition of ATGL caused very low plasma insulin concentrations upon fasting and increased insulin-mediated glucose clearance on chow and HFD [[62], [63], [64],^77^,^78^,^88^]. Insulin activates the transcription factor SREBP-1c that drives lipogenic gene expression. Yet, SREBP-1c transcript levels were markedly decreased in ATGL deficient WAT upon HFD [64,77] suggesting that insulin sensitivity appears to be selective for glucose metabolism but not lipogenesis. Together, these data point towards a downregulation of the master lipogenic transcription factors PPAR-γ and SREBP-1c as a metabolic compensatory mechanism to allow sufficient FA release from WAT.

ii) ATGL in energy balance

The obesity resistant phenotype upon genetic or pharmacological ATGL inhibition is partially also caused by reduced food intake. Obesity resistance and hypophagia were abrogated upon administration of the PPAR-γ agonist rosiglitazone [77,78]. The underlying mechanism(s) for these observations remain elusive but several scenarios are plausible. ATGL is expressed in the brain [89] and also Atglistatin® was detected at low levels in the brain after oral administration [54]. Hence, manipulating ATGL-mediated lipolysis in the brain may affect hypothalamic PPAR-γ signaling and thus appetite [90]. In a similar scenario, adipocyte lipolysis would generate a PPAR-γ (pro)-ligand, which via the circulation could affect central appetite regulation. However, also humoral factors like insulin and leptin affect central regulation of appetite [91] and these hormones are markedly altered upon genetic or pharmacological inhibition of ATGL [77,78].

Interestingly, pharmacological inhibition of ATGL points towards a defect in fat absorption that contributes to obesity resistance [78]. ATGL is expressed in enterocytes and a systemic or intestinal-specific genetic deletion of ATGL caused intestinal TG accumulation in mice [19,62]. Yet, TG secretion or lipid absorption were not altered upon loss of ATGL in enterocytes suggesting that ATGL is not essential for functional chylomicron secretion [19]. In line, deletion of the ATGL co-activator CGI-58 in enterocytes led to a pronounced TG accumulation in the proximal small intestine and a very moderate defect in TG secretion without altering fecal lipid secretion [92]. Together, the available data do not support a strong role of ATGL-mediated lipolysis in the small intestinal lipid absorption.

ATGL overexpression in adipose tissues also attenuated obesity upon HFD intervention [81]. In these mice, increased EE contributed to attenuated DIO [81]. In contrast, upon genetic or pharmacological inhibition of ATGL, no differences in EE were detected independent whether mice were housed at normal housing temperatures (21–23 °C) or at thermoneutrality (28–30 °C) where the need for thermoregulation is lowest in mice [64,77,78]. However, the obesity-resistant phenotype developed slowly and therefore it cannot be completely excluded that subtle differences in daily EE exist that are below detection limit using indirect calorimetry.

iii) ATGL and adipose tissue inflammation

Upon tissue expansion and increased FA mobilization from adipocytes, immune cells infiltrate WAT leading to a state of chronic inflammation [93]. In a vicious cycle, inflamed adipose tissue has been shown to exhibit increased FA release, partially due to elevated levels of the pro-inflammatory cytokine IL-6. This increase in FA release is supposed to be the main cause for obesity-associated metabolic derangements [94]. Pharmacological inhibition of ATGL completely blocked the IL-6 mediated increase in adipose tissue lipolysis indicating that ATGL is the main target for macrophage-induced lipolysis in adipose tissue [95]. On the other hand, genetic deletion of ATGL in adipocytes attenuated the infiltration of immune cells in WAT upon acute lipolytic stimulation indicating that ATGL-mediated lipolysis generates a signal for the recruitment of immune cells [64,96]. However, under non-stimulated conditions, adipocyte-specific ATGL knockout mice exhibited increased expression of pro- and anti-inflammatory marker genes in WAT and BAT on a chow and HFD [64]. In contrast, chronic pharmacological inhibition of ATGL using Atglistatin® reduced inflammatory marker gene expression and cytokine production in adipose tissues [78]. Possible explanations for this discrepancy between genetic deletion and pharmacological inhibition of ATGL include (i) the occurrence of adipocyte death upon total elimination of ATGL leading to an inflammatory response in adipose tissue, which does not happen upon transient ATGL inhibition [97]. Accordingly, the induction of NLRP3 and the formation of inflammasomes in BAT indicated that ATGL deficient brown adipocytes undergo “pyroptosis” leading to BAT inflammation, macrophage recruitment, and the formation of crown-like structures [98]. (ii) Residual ATGL activity and hence, functional PPAR-α signaling in Atglistatin® treated mice may be sufficient to counteract HFD-induced adipose tissue inflammation, which is not observed in genetic ATGL knockout mice with defective PPAR-α signaling [64,78]. And (iii), it cannot be excluded that Atglistatin® inhibits ATGL in macrophages. ATGL deficient macrophages showed impaired migration towards various chemo-attractants compared to wild-type. Additionally, macrophages deficient for ATGL exhibited impaired phagocytosis, which can been ascribed to reduced PPAR-β/δ activity and/or defective small Rho GTPase activation [99,74]. Moreover, reduced expression of the pro-inflammatory cytokines Gro1, IL-6, increased expression of mannose receptor 1, arginase 1, MCP 2, sphingosine kinase 1, as well as increased secretion of anti-inflammatory IL-10, and TGF-β argue for an anti-inflammatory phenotype of ATGL deficient macrophages [99].

### 3.3 Effect of ATGL on glucose homeostasis

Impaired glucose metabolism is one of the most important public health problems [100]. In the pathogenesis of insulin resistance (IR), several concepts assign a crucial role to lipids and in particular FAs. (i) According to Randle [101], increased availability of FAs promotes its oxidation and inhibits glucose oxidation. (ii) The concept of lipotoxicity introduced by Roger Unger [102] proposes that a lipid overload of non-adipose tissues and in particular key insulin target tissues causes tissue dysfunction. And (iii), lipids such as DGs, ceramides and/or acyl-CoAs are critical signaling molecules that may impair insulin signaling [[103], [104], [105]] but also promote insulin secretion [106]. Giving these key nodes of FAs, it is obvious that ATGL-mediated lipolysis will affect glucose homeostasis. This notion was confirmed by the initial characterization of systemic ATGL knockout mice which showed improved glucose tolerance and insulin sensitivity [62,88,107]. Moreover, these mice had increased glucose uptake in skeletal muscle, heart, and liver [62] and improved insulin signaling in skeletal muscle [88] suggesting that ATGL in these key insulin target tissues crucially affects whole-body glucose metabolism. Subsequent studies in tissue-specific ATGL knockout mice shed light to the enzymes' specific role in adipocytes, pancreatic β-cells, hepatocytes, and myocytes to influence glucose homeostasis.

i) Hepatic glucose production is controlled by adipocyte ATGL

Adipocyte-specific ATGL knockout mice exhibited reduced plasma FA levels. In accordance with the Randle hypothesis, these mice showed improved glucose tolerance on chow and HFD [35,62,64,77,95,107] as assessed by classic glucose and insulin tolerance tests (GTT and ITT, respectively) as well as by hyperinsulinemic-euglycemic clamp (clamp) studies. Remarkably, improved glucose homeostasis was independent of whether mice had increased obesity or were obesity resistant. Mechanistically, strong evidence suggests that ATGL in WAT affects substrate supply for gluconeogenesis in the liver. Using in vivo metabolomics, the group of Shulman [95] demonstrated that a low FA flux from WAT to the liver reduces hepatic acetyl-CoA levels and thus pyruvate carboxylase activity which subsequently suppresses hepatic glucose production (HGP). Furthermore, low WAT lipolysis reduces glycerol flux to the liver and thus further decreases gluconeogenesis. Similar to genetic deletion of ATGL, pharmacological inhibition of ATGL using Atglistatin® [54] – which primarily and transiently inhibits ATGL in adipocytes – positively affected glucose metabolism on chow and HFD [78,95]. Moreover, improved hepatic insulin signaling upon loss of ATGL in adipocytes likely contributes to decreased HGP [64]. Together, these studies demonstrate that ATGL-mediated lipolysis in WAT impacts glucose homeostasis via HGP.

In contrast to adipocyte-specific loss of ATGL, ATGL overexpression in adipose tissues (aP2-ATGL) is expected to increase FA flux and thus impair glucose metabolism. However, the contrary was observed. Despite increased adipocyte lipolysis, aP2-ATGL transgenic mice sustained normal plasma FA levels likely via increased recycling of FAs into TGs and/or increased FA oxidation in WAT/BAT [81]. Hence, aP2-ATGL transgenic mice showed increased glucose uptake in skeletal muscle and suppression of HGP.

ii) Pancreatic insulin secretion depends on white adipocyte and β-cell ATGL

FAs are critical for glucose-stimulated insulin secretion (GSIS) [108,109]. The current model suggests that exogenous FAs enter the β-cell via the FA receptors GPR40/FFAR1 [110], GPR120 [111], or CD36 [112]. Intracellularly, FAs are esterified into TGs and released from TGs by ATGL-mediated lipolysis to generate signaling molecule(s) which enable insulin secretion [106]. Several models support this concept of TG/FA cycling. (a) Systemic ATGL knockout mice showed extremely low plasma insulin concentrations during GTT [18,88]. Also adipocyte-specific ATGL knockout mice showed blunted plasma FA and insulin concentrations [63,64,113]. And (b) ATGL knockdown experiments in rat INS832/13 cells resulted in impaired fuel-stimulated insulin secretion [18]. Convincing proof for the role of ATGL in β-cells derived from two different mouse models [76,114]. Both studies used embryonic and inducible β-cell specific ATGL knockout mice that showed impaired GSIS and reduced plasma insulin concentrations [76,114]. However, the proposed molecular mechanisms and the metabolic consequences vary between the two models using different Cre mice. The group of Sul [76] – using the RIP-Cre and inducible RIP-CreER mouse – suggested that ATGL-mediated lipolysis is critical to liberate a lipid that activates PPAR-δ to promote mitochondrial function and ATP synthesis that is required for insulin secretion. These mice were glucose intolerant but had normal insulin sensitivity. The group of Prentki proposes an alternative mechanism mediated via MG signaling. According to their concept, ATGL-mediated lipolysis is required to provide MG species that are essential for insulin secretion [114]. Using the Mip-CreERT mouse, β-cell specific ATGL knockout mice had lower saturated MG species C16:0 and C18:0. In light of hypoinsulinemia, these mice had normal glucose tolerance suggesting improved insulin sensitivity. While the detailed signaling pathway awaits further investigation, current evidence shows that adipocyte and β-cell ATGL-mediated lipolysis are key for normal insulin secretion.

iii) Neither hepatocyte- nor myocyte-specific ATGL influences glucose metabolism

In contrast to white adipocytes and β-cells, the role of ATGL-mediated lipolysis in hepatocytes is less clear and available data on glucose homeostasis show divergent results. Adenovirus-mediated modulation of ATGL activity in the liver resulted in improved hepatic insulin signaling (Akt^Ser473^) upon overexpression [115] but not upon knockdown [116]. Yet, adenovirus-mediated ATGL knockdown improved plasma glucose and insulin concentrations as well as glucose tolerance [116]. In contrast, hepatocyte-specific ATGL knockout mice showed normal glucose and insulin tolerance as well as unchanged HGP assessed by pyruvate tolerance tests on chow and HFD [117]. All studies using either knockdown or gene deletion of ATGL showed progressive TG accumulation [[115], [116], [117]], increased DG content [116] but no changes in ceramides. Similarly, myocyte-specific ATGL knockout mice [118] exhibited normal glucose metabolism despite increased intracellular TG content on chow and HFD. These data suggest that hepatocyte and myocyte ATGL is not significantly influencing glucose homeostasis and indicates that neutral lipid accumulation per se in non-adipocytes is not detrimental.

iv) Glucose metabolism in NLSDM patients

The role of human ATGL in glucose metabolism is not conclusive. NLSDM patients do not show a homogenous metabolic phenotype (Table 2). To date, oral GTTs and clamp studies have been performed in few patients and suggest that loss of ATGL in humans affects insulin secretion but has no major impact on whole-body glucose metabolism [68,119,120]. In cases where type 2 diabetes developed, family history of diabetes existed and the pathology may have other causes than ATGL mutations.

### 3.4 The dichotomous role of ATGL in hepatic lipid metabolism and liver disease

The liver plays a central role in two global health threats. One is hypertriglyceridemia that is an important risk factor for cardiovascular disease. The second is non-alcoholic fatty liver disease (NAFLD) that embraces a disease continuum starting with TG accumulation in the liver (hepatic steatosis), which potentially proceeds to non-alcoholic steatohepatitis (NASH) that is accompanied by damaged liver cells, immune cell infiltration, and/or fibrosis, and may eventually cause cirrhosis and liver failure [121]. Therefore, to prevent hypertriglyceridemia and hepatic lipid accumulation, it is crucial to balance uptake, oxidation, re-esterification, and mobilization of FAs within the liver. Very recently, the role of different lipases in hepatic TG homeostasis has been reviewed in detail [122]. ATGL is expressed at low levels in liver parenchymal (hepatocytes) and non-parenchymal cells (hepatic stellate cells (HSC) and liver-resident macrophages/Kupffer cells) [1,123,124]. Furthermore, hepatic ATGL transcript levels are up-regulated upon fasting [125]. Thus, considering the key node of ATGL within the TG/FA cycle, ATGL-mediated lipolysis may have an important function in hepatic lipid homeostasis and progression of liver disease.

i) Adipocyte but not hepatocyte ATGL limits VLDL production

In liver, the hepatocyte is the major cell type accounting for >90% of total liver cells and is the site of FA turnover and VLDL synthesis. Initial characterization of systemic ATGL knockout mice revealed markedly lower plasma TG concentrations that suggested impaired VLDL secretion [62]. Yet, adenoviral-mediated ATGL knockdown and hepatocyte-specific genetic deletion of ATGL showed normal VLDL secretion in mice [73,117]. In contrast, adipocyte-specific ATGL knockout [64] and pharmacological ATGL inhibition [78] in mice reduced plasma TG concentrations demonstrating that the extrahepatic FA flux from WAT to the liver strongly affects VLDL secretion. These data again highlight the key role of ATGL-mediated lipolysis in WAT. In contrast to mice, the impact of human ATGL in lipoprotein metabolism is less understood. Overall, altered plasma lipoprotein content is not a common feature in NLSDM patients [55]. Normal [126] or increased [56,58,127,128] plasma lipoprotein concentrations have been reported for some NLSDM patients. Further investigations are required to better understand whether this inhomogeneity in hepatic lipid metabolism of NLSDM patients depends on the mutation or whether species-specific differences exist.

ii) Impact of adipocyte and hepatocyte ATGL on hepatic steatosis, inflammation, and disease

A constant elevated flux of FAs from adipose tissues to the liver is hypothesized to be the main cause for the development of hepatic steatosis [129]. Accordingly, adipocyte-specific ATGL or CGI-58 deletion as well as pharmacological ATGL inhibition [78] counteracts diet-induced hepatic steatosis. Hepatic PPAR-α signaling is also affected by impaired adipose tissue lipolysis as mice with an adipocyte-specific ATGL or CGI-58 deletion [64,130] and pharmacological ATGL inhibition [78] exhibit reduced hepatic PPAR-α target gene expression. Moreover, hepatic immune cell infiltration and inflammation was completely blocked in these mice indicating a delicate crosstalk between adipose tissue and liver via lipolytic products, presumably FAs [64,78,95,130]. This suggests that adipocyte lipolysis is a key determinant of the hepatic immune response. Of interest, also overexpression of ATGL in adipocytes improved hepatic steatosis, however, by using the aP2 promoter, ATGL may also be increasingly expressed in immune cells [81]. Taken together, these findings indicate that reducing adipose tissue lipolysis by targeting ATGL activity represents a promising tool for the treatment of inflammatory liver disease.

Next to adipocyte ATGL regulating FA flux to the liver, hepatocyte ATGL contributes to the turnover of TGs stored in cytosolic LDs. Adenoviral-mediated overexpression of ATGL in the liver increased FA oxidation and prevented hepatic steatosis [131]. In line, loss of ATGL in the liver either by adenoviral-mediated knockdown or by hepatocyte-specific gene deletion impaired FA oxidation and PPAR-α target gene expression subsequently causing progressive hepatic steatosis [73,117]. Mechanistically, Khan and coworkers [132] suggest that ATGL activates PPAR-α independent from ligand binding by activating SIRT1. The exact mechanism how ATGL activates SIRT1 remains elusive. Another pathway that may be affected by the loss of ATGL is the process of lipophagy. In contrast to lipolysis at neutral pH [122], lipophagy degrades lipids at acidic pH in a form of macro-autophagy and exerts a key role in hepatic TG metabolism [133]. Importantly, autophagy is induced by PPAR-α [134] and SIRT1 [135] and accumulating evidence suggests a crosstalk of cytosolic “neutral” lipolysis via ATGL and lysosomal “acid” lipolysis via lipophagy [136]. (i) ATGL interacts with LC3, an autophagosomal marker, in BAT and liver [9], (ii) chaperone-mediated lipophagy degrades LD-associated proteins allowing access of ATGL to its TG substrate for subsequent hydrolysis [137], and (iii) ATGL may directly promote autophagy/lipophagy via PPAR-α and SIRT1 signaling [138]. If and how “neutral” and “acidic” lipases act in concert has extensively been reviewed recently [136,139].

Loss of ATGL systemically or specifically in adipocytes or hepatocytes did not increase common markers for liver disease such as amino transaminases or inflammatory marker gene expression [78,107,117]. In addition, systemic ATGL knockout mice were protected from tunicamycin induced ER stress and hepatic inflammation. Mechanistically, this might be explained by the increased accumulation of “anti-lipotoxic” oleic acid compared to “lipotoxic” palmitic acid (PA) within LDs of ATGL deficient hepatocytes, which rescues these mice from PA-induced hepatic ER stress [142]. In contrast, hepatocyte-specific CGI-58 knockout mice developed steatohepatitis that was associated with increased plasma concentrations of amino transaminases and transcript levels of genes involved in inflammation and fibrosis [140]. Overall, these data suggest (i) an ATGL independent effect of CGI-58 in liver inflammation and (ii) that ATGL activity does not affect the liver's inflammatory signature under “non-stressed” conditions. However, ATGL appears to have an impact under stressed conditions. Upon induction of liver damage either by feeding methionine-choline-deficient diet (MCD) as a nutritional model of NASH or by endotoxin challenge using lipopolysaccharide for acute liver inflammation, hepatic inflammation aggravated in systemic ATGL knockout mice [141]. Interestingly, pharmacological PPAR-α agonist treatment using fenofibrate reverted hepatic steatosis, but only partially improved inflammation in MCD-fed ATGL knockout mice indicating a PPAR-α independent protective effect of ATGL on hepatic inflammation [141]. ATGL is also expressed in HSCs [5,124], which are the major site of RE storage within the body. Upon pathophysiological stimuli, HSCs activate to myofibroblasts and lose their RE stores [143]. Therefore, it is considered that a block of RE mobilization within HSCs can prevent liver fibrosis. Loss of ATGL in HSCs prevented the degradation of newly synthetized TG species [144] and RE mobilization from cultured primary HSCs [5]. Yet, hepatic RE content was not increased in systemic ATGL knockout mice suggesting a redundant enzyme system controlling RE mobilization. In line, systemic ATGL knockout mice fed a MCD diet showed increased fibrosis suggesting HSC activation [141]. Yet, to date, liver- and in particular HSC-specific ATGL knockout mice have not been tested in liver injury models. Interestingly, PNPLA3, the closest relative of ATGL within the PNPLA family, exhibits RE hydrolase activity in HSCs [145]. In contrast to ATGL, expression of a mutant variant of PNPLA3 (I148M) is associated with elevated hepatic RE and reduced hepatic retinol content implicating a possible role for PNPLA3 in retinol metabolism in vivo. [146].

In humans, ATGL and CGI-58 transcript levels were decreased in insulin-resistant NAFLD patients [147]. Fifteen NLSDM patients were reported to suffer liver dysfunction indicated by hepatomegaly and elevated plasma concentrations of amino transaminases (Table 2). However, no detailed information is available to date on liver inflammation or whether lipophagy is altered in NLSDM patients.

### 3.5 ATGL in cardiac and skeletal muscle function

i) Total loss of cardiomyocyte ATGL activity severely impairs cardiac function

The major energy fuel to guarantee constant ATP production for the heart is FAs. Delivery, uptake, mobilization, and oxidation of FAs are critical to maintain normal cardiomyocyte function [148]. Dysfunction of any of these processes may lead to altered substrate utilization and/or lipid deposition that can impair cardiomyocyte function, eventually causing a failing heart. Despite low mRNA expression levels, ATGL plays an essential role in the heart. In the mouse, genetic deletion of ATGL in the heart caused massive TG accumulation, progressive cardiomyopathy, and premature death [62,71,149]. In humans, ~50% of NLSDM patients develop progressive cardiomyopathy (Table 2). Most affected NLSDM patients are clinically silent until the age of 30 to 40 years. A key aspect for the progression of heart dysfunction is the functional consequence of the mutation and whether both alleles are affected. Two prominent examples support this notion. Example 1: Two patients have been reported with mutations affecting the catalytic dyad leading to an amino acid change of aspartic acid to glycine at position 166 (p.Asp166Ala) [67,150]. The mutated enzyme locates at the LD but is inactive. One patient is homozygous for the mutation, suffers from severe cardiomyopathy, and requires cardiac transplantation [67]. The second patient is heterozygous for the mutation and exhibits no severe heart problems [150]. Example 2: The missense mutation c.584C > T that causes the amino acid change proline to leucine at position 195 (p.Pro195Leu) within the N-terminal region of ATGL does not interfere with LD binding but abrogates ATGL activity. While two mutated alleles lead to severe cardiomyopathy, one wild-type allele appears sufficient to preserve residual lipase activity and thus delays disease progression (Table 2). In line with a correlation of ATGL activity and disease progression in humans, modulating ATGL activity in the mouse also correlates with the progression of cardiac dysfunction. For example, heterozygous ATGL knockout mice did not exhibit any cardiac defect and had normal life expectancy [62]. Furthermore, muscle-specific CGI-58 knockout mice showed delayed cardiomyopathy as compared to ATGL knockout mice [151]. Moreover, patients with mutations in the gene encoding CGI-58 have not been reported to develop cardiomyopathy [152]. Overexpressing G0S2 in the heart impaired cardiac lipolysis but had no effect on life span [125]. Importantly, pharmacological ATGL inhibition did not cause cardiac TG accumulation or heart dysfunction [78]. Therefore, in the future, it will be important to assess the biochemical impact of mutations in the human gene encoding ATGL in order to improve the patients' prognosis.

Mechanistically, TG accumulation within cardiomyocytes is expected to be a mechanical threat to the pumping heart. However, mouse models with impaired cardiac lipolysis such as overexpression of G0S2 or perilipin 5 exhibited similar TG levels in the heart as ATGL knockout mice but showed no signs of cardiac dysfunction [125,153]. Strong evidence demonstrates that ATGL-mediated lipolysis generates a (pro)-ligand for PPAR-α and promotes FA oxidation in the heart [71,154]. Subsequently, pharmacological PPAR-α activation in systemic ATGL knockout mice reversed the lethal phenotype. To date, two NLSDM patients have been treated with a PPAR-α agonist for 28 weeks [120]. The intervention improved fat oxidation and reduced tissue TG accumulation without major alterations in clinical parameters. Thus, further investigations are required to assess the impact of PPAR-α in NLSDM patients. A very recent report proposed the administration of tricaprin – a TG containing three medium-chain FAs (C8) – as functional food for NSLDM patients. Because of ATGL's substrate specificity for long chain FAs, treatment with tricaprin may prevent the massive accumulation of long-chain TGs in the heart [79]. In fact, feeding systemic ATGL knockout mice with tricaprin improved cardiac function [155]. The mechanism of tricaprin action is not known but it may serve as an alternative energy source or may be hydrolyzed by other lipases than ATGL. In vitro studies showed that long-chain TGs accumulate in NLSD fibroblasts, while short-/medium chain TGs are normally degraded suggesting a TG lipase specific for short-chain TGs [156]. The treatment of NLSDM patients with tricaprin awaits clinical trials. Together, the available murine and human data suggest that a complete block of ATGL-mediated FA release is detrimental for cardiac function, while modulating ATGL activity (by its co-regulators or by pharmacological intervention) obviously allows sufficient FA release to sustain FA supply as (pro)-ligands for PPAR-α activation.

ii) The impact of ATGL in protecting from heart failure

One of the hallmarks of metabolic disorders and certain drug treatments is increased cardiomyocyte TG content. Interestingly, several studies showed that cardiomyocyte-specific ATGL overexpression chronically reduced TG content in the heart and prevented cardiac dysfunction upon transverse aortic constriction [157], diabetes [158], obesity [159], and doxorubicin [160]. Chronically increased ATGL-mediated lipolysis in the heart reduced FA- and enhanced glucose oxidation. Upon diabetes or obesity, the expression of ATGL in the heart is induced indicating an adaptive yet insufficient response to the pathological increase in cardiac TG [158,159].

Remarkably, two very recent reports also indicate a crucial role for adipocyte ATGL in protecting the failing heart. Adipocyte-specific gene deletion and acute pharmacological inhibition of ATGL prevented the progression of heart failure induced by pressure-overload [161,162]. This concept suggests that reducing the FA flux from adipose tissue during the progression of heart failure prevents cardiac lipid remodeling presumably by stabilizing cardiomyocyte membrane integrity.

iii) NLSDM patients but not muscle ATGL knockout mice exhibit progressive myopathy

In murine skeletal muscle, ATGL is expressed at low but comparable levels to that in cardiac muscle [1] and increases during myogenesis [163]. Systemic or myocyte-specific ATGL knockout mice accumulate TGs in whole skeletal muscle, in particular within myocytes (intramyocellular TG, IMTG) [62,118] concordant with an important role of ATGL in muscle function. However, in strong contrast to the severe cardiac phenotype upon loss of ATGL in cardiomyocytes, the genetic deletion of ATGL in myocytes (systemically or tissue-specific) did not impair mitochondrial [118], contractile [164], or acute/peak exercise function [165]. Thus, modulating IMTG content is not pathologic per se. In contrast, adipocyte-specific ATGL limits the supply of FAs to the working muscle causing early fatigue during exercise [61,165].

Contrary to the benign muscle phenotype in the mouse, almost all NLSDM patients suffer from progressive myopathy with a high variability in severity (Table 2). In contrast to cardiac dysfunction, no clear correlation exists between ATGL activity/localization and the progression of myopathy. Histological analyses confirmed TG accumulation in muscle biopsies of NLSDM patients. CT and MRT analyses revealed that thighs and lower legs, shoulder girdle, and the paraspinal region are most affected and show fatty degeneration [128,166]. Due to lack of contractibility, muscle tissue is replaced by fat and connective tissue that further contributes to muscle weakness [167]. Accordingly, some NLSDM patients experience early fatigue [150,[166], [167], [168], [169], [170]]. To date, few exercise studies have been performed in NLSDM patients that are inconclusive but may indicate that exercise intolerance is caused by muscle weakness and – similar as in the mouse – by energy deficiency [171]. The mechanistic cause for the difference in the progression of myopathy between mice and men remains elusive. In humans, ATGL is exclusively expressed in type 1 (slow twitch oxidative) fibers [172]. In contrast, in the mouse, ATGL is expressed in all fiber types with highest transcript levels in oxidative fibers of type 2A (fast twitch oxidative) and at lower levels of type 2X and type 1 fibers [118]. Whether the difference in fiber type specific ATGL expression is disease causing awaits further investigations.

Data on ATGL expression in aged muscle – thus linking ATGL to sarcopenia – is controversial [163,173] but may suggest a protective function mediated via PGC-1α/PPAR-α. Myocyte-specific ATGL knockout mice did not show impaired PPAR-α signaling at the age of 10–30 weeks and observations from our laboratory indicate that also in aged (24 months) ad libitum fed mice ATGL deficiency does not impair muscle function. Whether reduced PPAR-α signaling contributes to myopathy in NLSDM patients is not known. Based on available clinical data, the median onset of myopathy in NLSDM patients was calculated to be 30 years [55] arguing for a progressive phenotype. It can be speculated that muscle atrophy in humans is caused by a reduced regeneration/differentiation capacity of ATGL deficient myofibers, whereas mice are less prone for muscle damage. Pharmacological PPAR-α activation in two NLSDM patients decreased IMTG content and moderately improved muscle strength [120]. Yet, these data are too preliminary to draw any solid conclusions.

### 3.6 ATGL in thermoregulation

Homeothermic vertebrates maintain their body temperature despite changing environmental temperatures, which allows continuous sustaining of normal body function. A highly specialized organ devoted to thermoregulation is BAT. BAT contains uncoupling protein 1 (UCP-1) which dissipates the mitochondrial proton gradient, thereby increases the electron flux and generates heat. Intracellular FAs are critical to induce UCP-1 and oxidative gene expression via PPAR-α [72,174], and to fuel UCP-1. Moreover, FAs activate UCP-1, although the exact mechanism is still not entirely understood [175]. UCP-1 mediated thermoregulation critically depends on the release of FAs within BAT via classical lipolysis and/or possibly via lipophagy [9]. While systemic HSL knockout mice were not cold sensitive [83,176], systemic ATGL knockout mice were severely cold sensitive suggesting a key role of ATGL for thermoregulation. Subsequent pharmacological inhibition studies in primary brown adipocytes corroborated that ATGL but not HSL is critical for maximal isoproterenol-induced mitochondrial respiration [177]. A definite in vivo proof for the role of ATGL-mediated lipolysis in thermoregulation, however, was only recently shown due to the availability of appropriate tissue-specific knockout mice. In contrast to in vitro evidence, BAT-specific ATGL or CGI-58 knockout mice were not cold sensitive [65,178]. Impaired BAT-specific ATGL-mediated lipolysis caused the expected TG accumulation in BAT, which morphologically rather resembled WAT. Yet, ATGL and/or CGI-58 deficient BAT maintained (i) normal UCP1 protein content, (ii) mitochondrial function, and importantly (iii) metabolic response upon β3-adrenergic receptor activation or cold, allowing normal thermoregulation. Upon loss of the ATGL co-activator CGI-58, the sympathetic tone increased and caused WAT browning [178]. Upon BAT-specific loss of ATGL, tyrosine hydroxylase protein expression – a readout for sympathetic innervation – was higher in BAT (unpublished observation) but WAT browning was not observed [65]. These data may suggest an ATGL independent function of CGI-58 in browning. The augmented sympathetic innervation may also explain the increased number of brown adipocytes in both mouse models. In contrast to BAT, a loss of ATGL or CGI-58 in BAT and WAT dramatically impaired thermoregulation upon fasting [35,65,178]. These data clearly demonstrate the importance of ATGL in WAT to deliver FAs towards BAT either directly or indirectly via the liver as VLDL-TGs [65] or acyl-carnitines [179]. Moreover, a very recent study expanded the critical role of ATGL-mediated FA release from WAT to promote insulin secretion to replenish the TG pool in BAT under acute catabolic thermogenic conditions [113]. An important question that remains to be answered is as “What ignites UCP-1?” [180]. It is undisputable that UCP-1 requires FAs [181] but the threshold of FA concentrations for activation is not known. Despite the loss of ATGL in BAT causing impaired TG hydrolase activity and reduced glycerol release, FA release was not blunted [65]. Next to ATGL, HSL exhibits little but some TG hydrolytic activity [182]. Therefore, it is feasible that a marginal FA release via HSL in brown adipocytes is sufficient to activate UCP-1. Recently, adipocyte-specific ATGL/HSL double knockout mice were generated which showed blunted lipolysis and even higher lipid accumulation in BAT than single ATGL or HSL knockout mice [183]. Double knockout mice were depleted of UCP-1 protein in BAT and had impaired thermoregulation if they were fasted during cold exposure – similar as for adipocyte-specific ATGL knockout mice. However, whether this cold sensitivity is due to impaired FA flux from WAT to BAT or within BAT is impossible to discriminate using this mouse model.

**Fig. 2:**
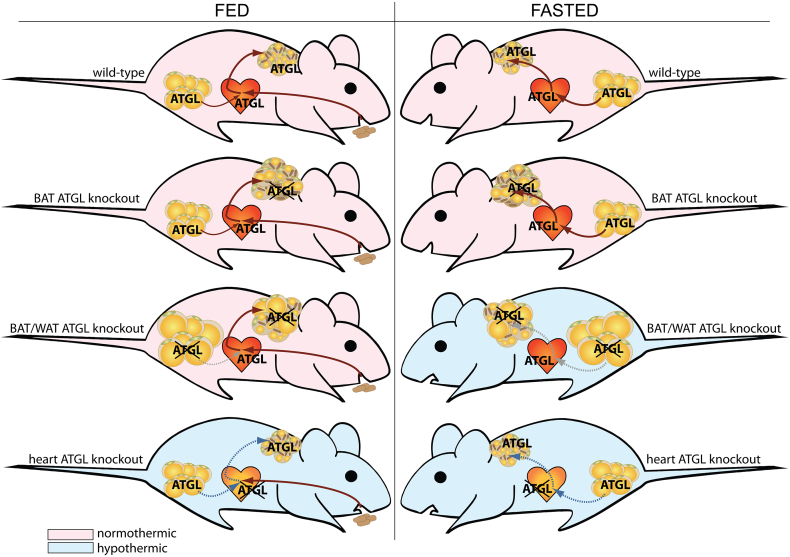
The role of ATGL in the regulation of cold-induced thermogenesis. Tissue-specific knockout mice have delineated the function of ATGL in maintaining body temperature upon cold exposure in the ad libitum fed state (FED) and upon food deprivation (FASTED). ATGL-mediated lipolysis in BAT is dispensable for cold-induced thermogenesis under fed and fasted conditions, but its activity in WAT becomes limiting upon caloric restriction (dashed grey lines). ATGL deficiency in the heart drastically impairs heart function and causes cold sensitivity independent of food supply or WAT and BAT lipolysis, presumably due to impaired hemodynamics (dashed blue lines). Knockout mice that have normal cold-induced thermogenesis are indicated in light red, mouse models with impaired cold-induced thermogenesis are indicated in light blue.

Enabling thermoregulation depends on UCP-1 but also on an inter-organ crosstalk. Particularly, during acute cold, it is critical that an organism can adopt muscle and heart function to the increased metabolic demand upon cold [184]. This fact has long been overlooked when discussing the role of ATGL in thermogenesis. Deleting ATGL specifically in the heart impaired heart function similar as to systemic ATGL knockout mice [62,149] and also caused cold sensitivity despite intact WAT and BAT lipolysis [65]. The cold sensitivity was dependent on progressive cardiac dysfunction and reversible upon restoration of heart function by re-expressing ATGL solely in the heart. These data show that upon loss of ATGL in the heart, mice were unable to maintain hemodynamics upon cold. The tissue-specific role of ATGL in cold-induced thermoregulation under ad libitum fed and fasted conditions is highlighted in Fig. 2.

### 3.7 ATGL in cancer and cancer-associated cachexia

**Fig. 3:**
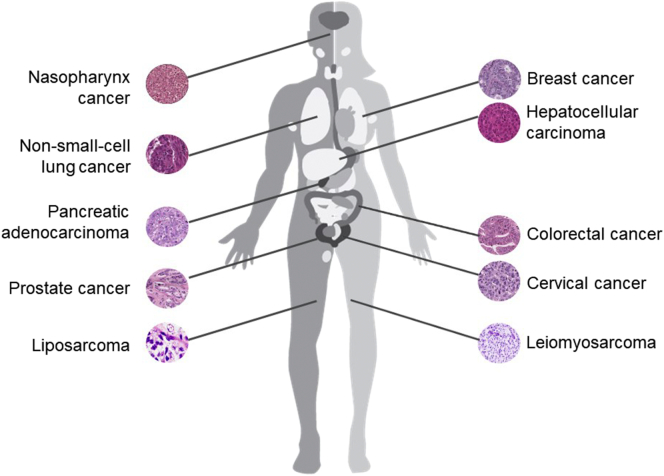
Association of ATGL with malignancies in (mice and) men. The pathogenesis and aggressiveness of several cancer types has been related to altered ATGL expression levels in mouse models and human patients. The scheme illustrates the ATGL association in different cancers.

Metabolic rewiring is a hallmark of cancer cells to sustain fast proliferation and survival, which are at the basis of tumor progression. Unlike well-defined changes in glycolysis, lipogenesis, and glutaminolysis, recent evidence has highlighted a deregulation of ATGL in tumorigenesis and the development of cancer-associated cachexia (CAC). However, current knowledge on how ATGL affects cancer metabolism and cancer pathogenesis is still elusive and controversial. The manifold associations of ATGL in malignancies that have been studied in mouse models and humans are depicted in Fig. 3.

i) Cancer

Recent studies in ATGL knockout mouse models and clinical samples have proposed a tumor suppressive feature of ATGL [14,183]. For example, systemic ATGL knockout mice exhibited spontaneous development of pulmonary neoplasia [14]. Moreover, mice lacking both ATGL and HSL specifically in adipocytes developed liposarcoma in BAT but not in WAT [183]. Consistently, a robust correlation between low ATGL protein expression in cancerous tissues and poor survival rate was reported in patients with non-small-cell lung cancer (NSCLC), pancreatic adenocarcinoma, and leiomyosarcoma [14]. Analyses of data from *The Cancer Genome Atlas* database further supports the notion that low ATGL transcript levels are a general event in at least 14 human malignancies [14]. Strikingly, a recent study reported reduced transcript levels of ATGL in a mouse model of induced hepatocellular carcinoma and in human biopsies [185]. Unpublished observations from our laboratory also suggest that tumorigenic progression correlates with reduced ATGL activity in different types of cancer. Although no definite molecular mechanism has been revealed yet, reduced ATGL expression may associate with certain oncogenes like Snail1, to block lipolysis in malignancy [186,187]. Furthermore, FoxO1, an established regulator of ATGL expression, is consistently reduced in many types of cancer [27,188,189]. Given this evidence, it is enticing to hypothesize that ATGL functions as tumor suppressor.

However, most of prior in vitro works have proposed that ATGL promotes cancer cell growth in a cell autonomous manner. shRNA-mediated knockdown of ATGL consistently impeded the proliferation of NSCLC cells, hepatic cancer cells, colon cancer cells, and prostate cancer cells [[190], [191], [192], [193]]. The underlying downstream mechanism has only been elucidated in NSCLC, where loss of ATGL caused TG accumulation and subsequent altered AMPK signaling leading to apoptosis [193]. Contrary to these reports, CRISPR/Cas9-mediated ATGL deletion contributed little to cancer cell proliferation or growth of tumor xenografts of colon and cervical cancer [47]. Besides its potential role in regulating cancer cell proliferation, ATGL was also suggested to modulate cancer aggressiveness. ATGL expression was found to be higher in aggressive breast cancer cells as compared with nonaggressive breast cancer cells as well as mammary epithelial cells [194]. Further experiments showed that ATGL upregulation in breast cancer was associated with a tumor microenvironment enriched in adipocytes, leading to a pro-oncogenic lipid network and subsequent invasiveness [194]. In contrast, pharmacological inhibition of ATGL or ATGL disruption by CRISPR/Cas9 led to a more aggressive phenotype in parallel with increased intracellular TG content of lung cancer cell lines [195]. This inconsistency of in vitro studies needs further clarification but may be due to different technologies and cancer types used for reducing ATGL activity.

The roles of ATGL's interaction partners particularly CGI-58, G0S2, and HILPDA in tumorigenesis also remain insufficiently explored. Recent studies identified CGI-58 as a potential tumor suppressor showing that the loss of CGI-58 increased the propensity for tumor growth of prostate cancer and colon cancer [190,192,196]. Notably, ATGL independent mechanisms were held responsible for CGI-58 dependent cancer phenotypes including the regulation of the AMPK axis and the interaction with the autophagy gene BECN1. As a protein participating in cell cycle regulation, G0S2 acts as a tumor suppressor. One study suggested that G0S2 deficiency promotes the growth and motility of NSCLC cells by derepressing ATGL activity [193]. However, Yim et al. [197,198] reported that the tumor suppressive role of G0S2 was ATGL independent. Moreover, a recent study proposed that HILPDA but not G0S2 downregulated ATGL activity of hypoxic cancer cells [47]. HILPDA exerted an oncogenic function by neutralizing the tumor suppressive role of ATGL under hypoxic conditions, whereas disruption of ATGL and/or HILPDA incurred little changes for cancer cell growth under normoxia [47]. Although still preliminary and incomplete, these results highlight a previously underestimated role for ATGL and its interaction partners in cancer cell metabolism and tumorigenesis. Further investigations are required to better characterize their roles in the development, growth, and aggressiveness of different cancers.

ii) Cancer-associated cachexia

Twenty percent of all cancer deaths are not caused by the cancer burden itself but by the associated wasting syndrome CAC [199]. Patients suffering CAC are characterized by an unintended loss of body weight due to WAT and skeletal muscle atrophy [200]. A panel of studies reports that the expression and activity of ATGL is induced upon CAC in adipose tissues of genetically manipulated mice and individuals, and contributes to the wasting process in adipose tissues [[201], [202], [203], [204]]. Importantly, loss of ATGL in mice partially preserved WAT mass in two murine cancer models [201]. Mechanistically, it is widely accepted that increased concentrations of many cytokines, such as TNF-α, IL-6, leukemia inhibitory factor, parathyroid-hormone-related protein, and other cachexia inducing factors trigger the catabolic events in fat depots [202,203,205,206]. However, the direct cause for increased ATGL expression and activity in CAC remains largely unexplored and requires further investigation [207].

### 3.8 Other tissues

i) ATGL in immune cells

In humans, a key diagnostic marker for NLSDM is Jordans' anomaly namely the accumulation of LDs in leukocytes [152]. Therefore, it is tempting to speculate that ATGL impacts immune cell function. Indeed, the Kratky laboratory showed that macrophages deficient for ATGL exhibited impaired phagocytosis, which can been ascribed to reduced PPAR-β/δ activity and/or defective small Rho GTPase activation [99,74]. Moreover, genetic or pharmacological inhibition of ATGL in immune cells led to an accumulation of arachidonic acid (AA), a precursor for pro- and anti-inflammatory signaling molecules, in their TG pool thereby sequestering an important source for immuno-modulatory precursors in neutrophils and mast cells [15,16]. Hence, ATGL deficiency especially in granulocytes and lymphocytes may impair infection defense. In humans, recurrent infections have been reported in a family of 6 individuals [126]. However, these persons were related and heterozygous mutant carriers. Thus, these infections may have other causes than mutations in the ATGL gene.

ii) ATGL in male reproduction

Systemic deletion of ATGL leads to a pronounced TG accumulation in testis [62]. A recent study reported lower total and motile sperm concentrations upon systemic ATGL knockout suggesting impaired spermatogenesis and sperm maturation [208]. However, systemic ATGL knockout with rescued heart function [71,77] were bred using heterozygous females and homozygous males and produced siblings (unpublished data). Thus, these observations suggest that upon loss of ATGL sperm maturation may be delayed but mice are not sterile. More likely, energy insufficiency and cardiomyopathy contribute to low or no reproducibility of males with a systemic ATGL deletion. In humans, no reports about male infertility of NLSDM patients are available.

iii) ATGL in the skin

In the 1960s, first cases of ichthyosis accompanying NLSD were reported [209]. Some years later, NLSD associated with ichthyosis was termed Chanarin-Dorfman syndrome (CDS) and characterized by TG accumulation in most tissues of the body including blood cells (Jordans' anomaly), skin, liver, skeletal muscle, and the heart [210]. In 2001 and 2007, the group of Fischer identified mutations in the genes encoding CGI-58 and ATGL to be responsible for CDS [59,211]. However, in contrast to the classical ichthyotic phenotype of CDS, NLSDM patients who carry mutations in the human gene encoding ATGL do not develop ichthyosis (Table 2, [59]). Studies in mice verified the observations in humans. In contrast to systemic or epidermis-specific CGI-58 gene deletion, systemic ATGL knockout mice do not develop a defective skin permeability barrier despite epidermal TG accumulation suggesting an ATGL-independent function of CGI-58 in the skin [212,213]. In fact, very recently CGI-58 has been shown to stimulate ω-O-acylceramide synthesis in the skin that depends on protein interaction with PNPLA1, a close homologue of ATGL [214]. The role of PNPLA1 in ω-O-acylceramide synthesis and skin barrier function is discussed in detail within this Special Issue by Hirabayashi et al. [215].

iv) ATGL in neuronal signaling

ATGL is expressed in the murine neuronal system. In the brain, ATGL transcripts are found particularly in ependymal cells, the choroid plexus, and the hippocampus [89]. Systemic loss of ATGL resulted in TG accumulation in these brain compartments that are involved in exchange processes with the periphery such as the brain-cerebrospinal and the blood-brain barrier. It may be speculated that FAs released by ATGL have an important function in signaling and survival/regeneration of neuronal cells. Owing to the specificity of ATGL for long-chain FAs and to its phospholipase activity, ATGL hydrolyzes phospholipids that are rich in docosahexaenoic acid (DHA) and AA. These long-chain FAs have a critical role in hippocampal long-term potentiation, learning ability, and cognitive function [89]. However, to date only one NLSDM patient has been described to suffer mental retardation (Table 2) and no animal studies have been performed to investigate whether modulating ATGL activity affects brain function.

In the sensory nervous system, ATGL transcripts were detected in the cornea [216], the retinal pigment epithelium, and at lower levels in the inner segments of the photoreceptors, and retinal ganglion cell layer of the native retina in humans and rats [6]. In these cells, ATGL protein is proposed to serve as receptor for PEDF. Subramanian et al. [49] suggest that binding of PEDF to ATGL increases its phospholipase activity and stimulates a signaling cascade that involves DHA leading to reduced apoptosis of retinal cells and increased corneal nerve regeneration [49,216]. In cornea, pharmacological inhibition of ATGL using Atglistatin® decreased the release of DHA leading to a decelerated regeneration of corneal nerves after injury. Although expressed in human optical tissue, there is no report on an impairment of eyesight in NLSDM patients (Table 2). Interestingly, PEDF has been shown to have a neuroprotective effect on motor neurons from spinal cord [217], on cerebellar granule neurons [218], and on neurons from the hippocampus [219]. Hence, it is tempting to speculate that PEDF exerts its neurotrophic effects via ATGL also in the central nervous system.

## 4 Concluding remarks and perspectives

**Fig. 4:**
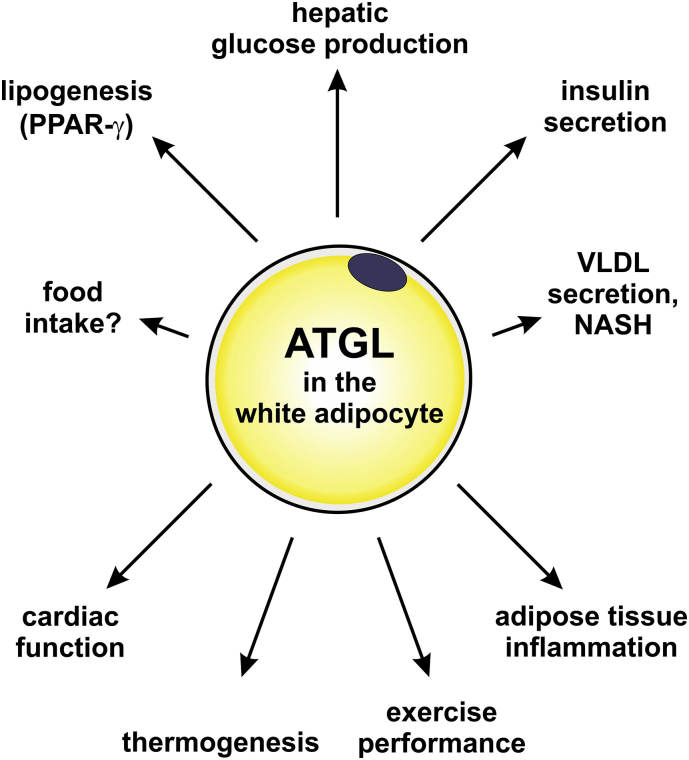
ATGL-mediated lipolysis in the murine white adipocyte affects numerous physiological processes. This simple graph illustrates the manifold physiological functions that are affected by white adipocyte ATGL-mediated lipolysis.

Genetic mouse models with tissue-specific overexpression or deletion of ATGL have provided key insights into the importance of ATGL-mediated lipolysis in mouse physiology. In particular, ATGL-mediated FA flux from white adipocytes influences numerous physiological processes in the mouse that are summarized in Fig. 4. Thus, inhibiting ATGL specifically in white adipocytes appears to harbor therapeutic potential. A pharmacological approach using the ATGL inhibitor Atglistatin® has recapitulated many phenotypes of genetic mouse models. Most importantly, however, the detrimental cardiac phenotype of loss-of-function models was not observed upon Atglistatin® treatment. However, despite >40 NLSDM patients, the impact of ATGL in regulating plasma FA concentrations is less clear in humans. In the future, to validate ATGL's potential serving as a drug target, it will be important to study humanized mouse models and to develop an inhibitor specific for human ATGL but also to clarify the role of ATGL in cancer progression.

## Abbreviations

AA: arachidonic acid
ATGL: adipose triglyceride lipase
BAT: brown adipose tissue
CAC: cancer associated cachexia
CDS: Chanarin-Dorfman syndrome
CGI-58: comparative gene identification-58
clamp: hyperinsulinemic-euglycemic clamp
DG: diglyceride
DHA: docosahexaenoic acid
DIO: diet-induced obesity
EE: energy expenditure
FA: fatty acid
G0S2: G0/G1 switch gene 2
GTT: glucose tolerance test
HFD: high-fat diet
HGP: hepatic glucose production
HILPDA: hypoxia-inducible LD-associated protein
HSL: hormone-sensitive lipase
GSIS: glucose-stimulated insulin secretion
IMTG: intramyocellular triglyceride
IR: insulin resistance
ITT: insulin tolerance test
LD: lipid droplet
MCD: methionine-choline-deficient diet
NAFLD: non-alcoholic fatty liver disease
NASH: non-alcoholic steatohepatitis
NLSD(M): neutral lipid storage disease (with myopathy)
NSCLC: non-small-cell lung cancer
MG: monoglyceride
PA: palmitic acid
PEDF: pigment epithelium-derived factor
PKA: protein kinase A
PNPLA2: patatin-like phospholipase domain containing 2
PPAR: peroxisome proliferator-activated receptor
RE: retinyl ester
TG: triglyceride
UCP-1: uncoupling protein 1
WAT: white adipose tissue

## Transparency document

Transparency document. Image 1
